# Dual-Laser-Induced Breakdown Thermometry via Sound Speed Measurement: A New Procedure for Improved Spatiotemporal Resolution

**DOI:** 10.3390/s20102803

**Published:** 2020-05-14

**Authors:** Shen Li, Wubin Weng, Chengdong Kong, Marcus Aldén, Zhongshan Li

**Affiliations:** 1 Division of Combustion Physics, Lund University, P.O. Box 118, 22100 Lund, Sweden; shen.li@forbrf.lth.se (S.L.); wubin.weng@forbrf.lth.se (W.W.); marcus.alden@forbrf.lth.se (M.A.); zhongshan.li@forbrf.lth.se (Z.L.); 2 Institute of Thermal Energy Engineering, School of Mechanical Engineering, Shanghai Jiao Tong University, Shanghai 200240, China

**Keywords:** dual-laser-induced breakdown thermometry, laser-induced acoustic wave, measurement of acoustic wave, high-temperature measurement, combustion atmosphere

## Abstract

Measurement of acoustic waves from laser-induced breakdown has been developed as gas thermometry in combustion atmospheres. In the measurement, two laser-induced breakdown spots are generated and the local gas temperature between these two spots is determined through the measurement of the sound speed between them. In the previous study, it was found that the local gas breakdown can introduce notable system uncertainty, about 5% to the measured temperature. To eliminate the interference, in present work, a new measurement procedure was proposed, where two individual laser pulses with optimized firing order and delay time were employed. With the new measurement procedure, the system uncertainty caused by local gas breakdown can be largely avoided and the temporal and spatial resolutions can reach up to 0.5 ms and 10 mm, respectively. The improved thermometry, dual-laser-induced breakdown thermometry (DLIBT), was applied to measure temperatures of hot flue gases provided by a multijet burner. The measured temperatures covering the range between 1000 K and 2000 K were compared with the ones accurately obtained through the two-line atomic fluorescence (TLAF) thermometry with a measurement uncertainty of ~3%, and a very good agreement was obtained.

## 1. Introduction

Nowadays, a pulsed laser can be easily focused to breakdown gases, which generates heat, optical emission, and acoustic emission. The optical emissions are used in the conventional laser-induced breakdown spectroscopy (LIBS) technique to realize in-situ element measurements in various application fields [1,2]. While in recent years, the acoustic emission from the laser-induced breakdown has gained more attention and been successfully applied for different purposes, such as the understanding of the propagation mechanism of the laser-generated shock wave, the nonlinear absorption of laser in the gas, and the detection of the particle size and concentration [2]. More recently, the acoustic emission has been employed for in-situ gas temperature measurement. 

In the study of combustion, the temperature is one of the most crucial parameters. Lots of methods have been developed and employed for obtaining accurate temperature measurements in combustion atmospheres. A conventional simple method is the thermocouple-based thermometry, which is intrusive and requires corrections of radiative and conductive heat losses. Non-intrusive laser-based techniques have been developed for temperature measurements, such as Rayleigh scattering thermometry (RST) [3,4], laser-induced fluorescence (LIF) thermometry [5], coherent anti-strokes Raman spectroscopy (CARS) [6,7], tunable diode laser absorption spectroscopy (TDLAS) [8], and two-line atomic fluorescence thermometry (TLAF) [9]. Those methods have their own peculiarities and disadvantages. Rayleigh scattering is an incoherent and non-species-selective technique. For accurate temperature measurement, the gaseous species compositions in the probed volume and the Rayleigh cross-sections of individual species must be considered. Furthermore, the Rayleigh scattering signal is sensitive to stray lights from the background. TDLAS is a line-of-sight technique, which is difficult to measure spatial variation in temperature. The LIF method is limited by quenching effects. The CARS method is usually a point measurement technique, and its optical system is more complicated than other methods. The disadvantage of TLAF is that an atomic species, not normally present in the flame, has to be introduced which may add complexities to the experimental setup. These laser-based techniques such as CARS, LIF, RST, and TLAF are usually expensive as the used lasers need to be powerful, stable, or wavelength-tunable. Recently, the laser-induced breakdown thermometry technique (LIBT) has been proposed to be robust and simple with a compact experimental setup. 

There are several methods for the laser-induced breakdown thermometry. One approach used the peak pressure of the acoustic emissions from laser-induced breakdown, proposed by Wu et al. [10]. Given a fixed laser pulse energy and distance, the peak acoustic pressure is determined by sound speed, gas density, and the specific heat. As all the parameters are functions of gas temperature, the peak pressure can be correlated with the local gas temperature [11]. In practice, the recorded acoustic signals need to be fitted with a power-law to the temperature measured by a thermocouple to obtain the calibration curve. Although thermocouples are reliable, errors of the temperature measurements can occur for various reasons, and the calibrated temperature range is limited by thermocouples. Kiefer et al. proposed another approach for gas thermometry, which is based on the temperature dependency of the breakdown threshold of laser pulse energy [12]. It also requires a careful calibration as well as a correction of chemical composition effects. Recently, Lee et al. proposed the third approach which is based on the laser-induced breakdown acoustic wave propagation speed measurement [13]. The temperature accuracy of Lee’s approach is relatively low with a systematic uncertainty of ~5% for flame temperature measurement [14]. This is because that, in Lee’s measurement, a single laser was split to generate two laser-induced breakdowns simultaneously. To measure the sound speed between the two breakdown spots, the transmission time between the breakdown spots was recorded using a sound detector. It was noted that the sound waves generated by the breakdown spot far from the detector would pass through the breakdown spot close to the detector. The passage before the cooling down of the breakdown spot which takes at least 100 µs could inevitably introduce a large system error [15]. 

In this work, two individual lasers with optimized firing order and delay time were employed to improve the spatiotemporal resolutions and the accuracy of the laser-induced breakdown thermometry. A calibrated hot environment supported by a multi-jet burner was utilized to justify the applicability to temperature measurements in combustion environments.

## 2. Fundamental Theory of *LIBT*

The speed of sound in an ideal gas can be expressed as

(1) $$V_{0}={\left(\frac{kR_{0}T}{M}\right)}^{\frac{1}{2}}$$

where *k* is the specific heat ratio, *R*_0_ is the ideal gas constant, *M* is the molecular weight, and *T* is the temperature [16]. Taken the specific heat ratio for the mixture as the mole weighted specific heat ratio and the molecular weight of the mixture *M* as the average mole weights of mixtures, *k* can be expressed as (For a gas mixture with ni moles of the i-th gas):

(2) $$k=\frac{\sum_{i}C_{pi}x_{i}}{\sum_{i}C_{vi}x_{i}}=\frac{\sum_{i}n_{i}C_{p,i}}{\sum_{i}n_{i}C_{v,i}}=\frac{\sum_{i}n_{i}\frac{k_{i}}{k_{i}+1}}{\sum_{i}n_{i}\frac{1}{k_{i}+1}}$$

and *M* is:

(3) $$M=\sum_{i}M_{i}x_{i}$$

In Equation (2), *C_v,i_* is the specific heat capacity at constant volume. *C_p,i_* is the heat capacity at constant pressure. Combing Equations (1), (2) and (3), the speed of sound can be expressed as

(4) $$V=\sqrt{\frac{\sum_{i}C_{pi}x_{i}}{\sum_{i}\left(C_{pi}-R_{0}\right)x_{i}\sum_{i}M_{i}x_{i}}\cdot R_{0}T}=\sqrt{\left(\frac{1}{\sum_{i}x_{i}\frac{1}{k_{i}+1}}-1\right)\cdot \frac{R_{0}T}{\sum_{i}M_{i}x_{i}}}$$

where *x_i_* is the mole fraction, *M_i_* is the molecular weight of species i. To determine the temperature in Equation (4), both the sound of speed in the gas mixture and the mole fractions of the species in the probed volume are to be measured or estimated. In our study, the specific heat ratios and the molecular weights of species are derived from the CHEMKIN-Pro software.

## 3. Experimental Setup 

**Figure 1:**
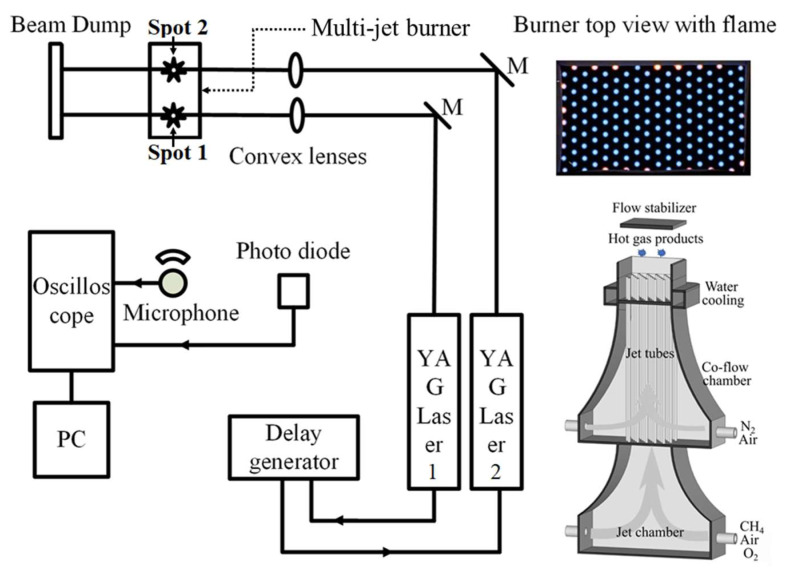
Experimental setup for dual-laser-induced breakdown acoustic wave measurement.

Figure 1 shows a schematic of the experimental setup. Two Nd:YAG lasers (Quantel, Les Ulis, France) with second harmonic units(Quantel, Les Ulis, France) were used to generate two 532 nm laser beams. The laser beams had a pulse duration of 6 ns at 10 Hz and a diameter of ~9 mm. They were focused 5 mm above the laminar flame burner by two convex lenses with a focal length of 15 cm, as shown in Figure 1. The minimum pulse energy for LIBT depends on the focal length of the lens and the gas temperature. Usually, the threshold for laser-induced gas breakdown increases with temperature. The produced acoustic signal intensity grows with pulse energy. In this work, the laser energy was set to be around 100 mJ per pulse to breakdown the hot gas with a laser fluence at the focal point of around 2.426 × 10^17^ J/m^2^. This chosen pulse energy is larger than the local threshold to ensure the gas breakdown when the laser energy fluctuates from pulse to pulse. The laser-induced acoustic signal is captured by a microphone with a high dynamic frequency range (20 Hz–20 kHz). The microphone is positioned at 29.52 cm away from the closer laser-induced breakdown spot (Spot 1) and aligned with the two breakdown spots, as shown in Figure 1. The signal from the microphone was acquired by an oscilloscope (Picoscope 4424, Pico Technology Ltd, Eaton Socon, UK) with a sampling rate of 80 MHz. A photodiode was used to record the moment of the breakdowns. Based on the time delay between the signals from the photodiode and the microphone, the sound speed between these two breakdown spots can be determined for temperature calculation. To avoid any influence on the local temperature from the breakdown, the laser for generating the far-away breakdown (i.e., Spot 2) is fired firstly, and after a certain delay time, the other laser is triggered to produce the other breakdown spot (i.e., Spot 1). The delay time was precisely controlled by a digital delay generator (DG535, Stanford Research Systems, Sunnyvale, CA, USA). 

The measurement positions are symmetrically located in the central zone of the hot flue gas provided by a laminar flame burner, namely a multijet burner [17]. The burner has an outlet size of 60 mm × 100 mm and consists of two chambers for the jet-flow and the co-flow, separated by a divider. The jet flow, which is mainly a mixture of CH_4_/air/O_2_, was introduced into the jet-flow chamber through four inlets to generate premixed flames anchored on each jet tubes as shown in Figure 1. Each jet tube was evenly surrounded by the co-flow with a composition of air/N_2_. By varying the gas compositions of the jet-flow and the co-flow, the temperature and the composition of the hot fuel gas mixture can be controlled. In the present work, the temperature was varied in the range from 1000 K to 2000 K and the global equivalence ratio was varied from 0.63 to 1.32. The flow speed of the hot flue gas at the outlet of the burner was around 1 m/s, which is much smaller than the sound speed. All gas supplies were controlled by mass flow controllers (Bronkhorst High-Tech B.V., RUURLO, Netherlands) with an accuracy of ± 0.8% and the reading value plus ± 0.2% of the full-scale value. The high-temperature cases applied in the present study are summarized in Table 1 with the temperature measured by TLAF with indium atoms.

The acoustic emissions due to the laser-induced breakdown are investigated in the hot flue gas with different global equivalence ratios and different temperatures. The measurements were repeated five times for each flame condition, and 100 samples were collected for each measurement. Therefore, the value of the average sound speed between the two breakdown spots was determined, and temperatures of the hot flue gases were derived.

The temperatures obtained by DLIBT was compared with the one obtained by TLAF. Detailed TLAF technique with Indium seeding has been systemically described by Borggren et al [9]. In this study, a similar setup has been used, mainly including three parts: the laser, the seeding system, and the imaging system. An indium chloride (InCl_3_) solution was added to the seeding system. Two external cavity diode lasers (Model DL10pro and DL100, TOPTICA Photonics AG, Graefelfing, Germany) were separately controlled by two analog control units in the laser system, each including a temperature control module (DTC 100, TOPTICA Photonics AG, Graefelfing, Germany) and a current control module (DCC 110, TOPTICA Photonics AG, Graefelfing, Germany) Two continuous-wave laser beams at 410 nm and 451 nm were produced with a power of 5 mW and a beam size of 1 mm^2^. These two laser beams were overlapped by a dichroic mirror and passed through the hot flue gas at a height of 5 mm above the burner outlet. Two different lower levels of the indium atom are excited to a common upper level, using the transition 5^2^P_1/2_ → 6^2^S_1/2_ and 5^2^P_3/2_ → 6^2^S_1/2_, and the fluorescence at 451 nm and 410 nm were captured by an ICCD camera (Model PI-MAX3, Teledyne Princeton Instruments, Trenton, NJ, US) through a band-pass filter. Thus, a temperature profile above the burner was obtained by evaluating the fluorescence signal.

Moreover, the adiabatic flame temperature at different global equivalence ratio was obtained using the chemical equilibrium model in CHEMKIN software (Reaction Design, San Diego, CA, USA) [18] with the GRI-3.0 mechanism [19] to compare with the experimental results.

## 4. Results and Discussion

**Figure 2:**
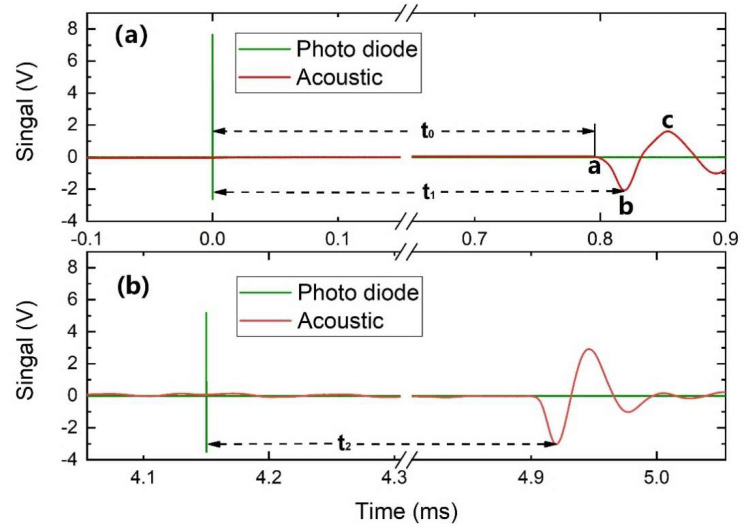
Time-domain photodiode signal and the laser-induced breakdown acoustic signal from the far-away laser (spot 2) (**a**) and the closer laser (spot 1) (**b**) in the flame with the equivalence ratio equal to 0.67. The distance of the microphone to the closer laser is 29.52 cm.

Figure 2 shows the time domain photodiode signal and the laser-induced acoustic signal with a laser-induced breakdown in the hot flue gas provided by the flame with a global equivalence ratio at 0.67. In this work, the arrival time is defined as the time of the acoustic wave traveling from the laser breakdown spot to the microphone detector with the first negative bump of the acoustic signal. The arrival time of the far-away laser-induced breakdown (Spot 2) and the closer laser-induced breakdown (Spot 1) was indicated as t1 and t2, respectively, as shown in Figure. 2. Thus the time of flight between two breakdown spots can be expressed as Δt = t1–t2. In fact, as shown in Figure. 2, different turning points (i.e., Points a, b, c in Figure 2a can be used to calculate Δt. It is found that when the first negative peak (i.e., Point b in Figure 2a is chosen, the obtained Δt has less standard deviation, so is more accurate for the measurement of sound speed. It is noteworthy that the high-energy pulse laser system always has timing jitter. However, by obtaining the optical signal and the acoustic signal simultaneously, the impacts of timing jitter on the measured arrival time can be avoided. It implies that this technique do not require small time jittering of the adopted pulsed laser.

**Figure 3:**
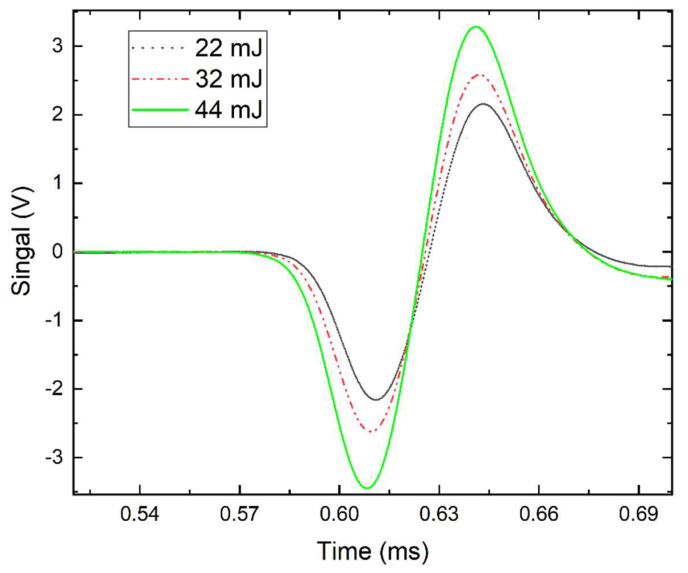
Acoustic wave signals from the laser-induced breakdown Spot 2 under different laser pulse energy.

To minimize the uncertainties in the laser-induced breakdown thermometry, some specific procedures are proposed. Firstly, the laser pulse energy at each breakdown spot has been carefully adjusted using a variable attenuator to the same value. When a laser-induced breakdown occurs, a shock wave is firstly created and then transfers to an acoustic wave within a few millimeters [20,21]. Therefore, the accuracy of the measured acoustic speed can be influenced if the whole wave propagation period is considered as the acoustic wave propagation. When the laser breakdown occurs in a static ideal gas medium, the characteristic radius Ra_0_ which describes how far the shockwave, generated by the laser-induced breakdown can travel, is expressed as Equation (5).

(5) $${\mathrm{Ra}}_{0}=1.6374\times {\left(\frac{E}{P_{0}\gamma }\right)}^{\frac{1}{3}}$$

where P_0_ is the initial pressure, *E* = α*E*_0_, *E*_0_ is the laser breakdown energy and α is the medium constant. When the shockwave propagates further than Ra_0_, it transfers into a mechanical wave in the acoustic speed [21,22]. To further understand this phenomenon, only one laser was used to breakdown the gas at room temperature. The arrival time becomes longer when the distance between the breakdown spot and the microphone is increased. The derived acoustic wave speed is about 470 m/s within 10 mm, which is 1.4 times higher than the speed of sound. As the microphone is moved further away from the breakdown spot from 100 mm to 220 mm, the acoustic wave propagation speed drops to the speed of sound, since the shockwave becomes a mechanical acoustic wave at the speed of sound beyond a certain distance. The speed derived from the fitting data is 346 m/s. According to Equation (5), the characteristic radius Ra_0_ depends on the laser breakdown energy, which finally influences the acoustic wave speed. Figure 3 shows the acoustic signal from the laser-induced breakdown under different laser pulse energies. It illustrates that the arrival time decreases slightly, and the amplitude of the signal increases with the laser pulse energy increase. It indicates that besides the distance between the microphone and the breakdown spot, the laser pulse energy will influence the arrival time as well. Thus, the pulse energy of the two lasers is better to be the same to form the breakdown region with the same characteristic radius Ra_0_ to avoid the contribution of the nonlinear shockwave propagating regions to Δt as the influence from the nonlinear shock wave is offset. The calculated time duration between two breakdown spots is reliable for further temperature derivation. It should also be mentioned that even though the pulse energy impacts Δt, its influence is marginal according to Equation (5) when the laser pulse energy fluctuates from pulse to pulse with an uncertainty of 4%.

Secondly, for accurate measurement of temperature, the distance between the dual laser-induced breakdown spots need to be accurately measured. We propose to calibrate this distance by using the same setup at the laboratory conditions (23 °C, 1 atm), where the sound speed is known. When Δt between two breakdown spots is measured with a fixed laser pulse energy of 44 mJ in the air, the distance can be determined by Δt times the speed of sound at 23 °C (345 m/s), which is estimated to be 39.01 mm when Δt is 112.6 μs. The temperature in the laboratory is measured by a digital thermometer with an uncertainty of ± 0.5 °C. It results in an uncertainty of ± 0.3 m/s for the measured sound speed and finally, an uncertainty of distance is ± 0.034 mm. 

Thirdly, the DLIBT benefits from a two-laser setup where the time delay between lasers can be manipulated. Meanwhile, by choosing the right firing order of two laser pulses, that the far-away laser-induced breakdown (Spot 2) is generated firstly (normal sequence), it can avoid one of the acoustic waves passing through the heated region by another temporally.

**Figure 4:**
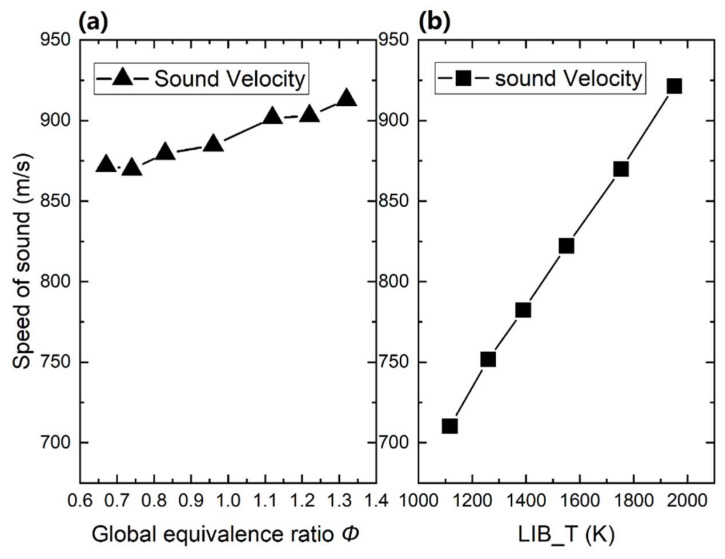
Calculated sound speed as a function of global equivalence ratio at 1800 K (**a**) and calculated speed of sound as a function of temperature at a global equivalence ratio equals to 0.65 (**b**)**.**

With the obtained speed of sound, the temperature of the hot flue gas can be obtained. However, based on Equation (4), not only the temperature but also the gas composition influences the speed of sound. Figure 4 shows the calculated speed of sound in each flame case as a function of the global equivalence ratio and gas temperature. As the global equivalence ratio varies from 0.67 to 1.3 with a constant temperature of around 1800 K, the sound speed changes between 870 m/s and 913 m/s, while the sound speed increases dramatically from 710 m/s to 921 m/s with the temperature increased from 1159 K to 1990 K with a global equivalence ratio around0.7. For the cases employed in the present work, the derived sound speed is more sensitive to the temperature than species composition.

**Figure 5:**
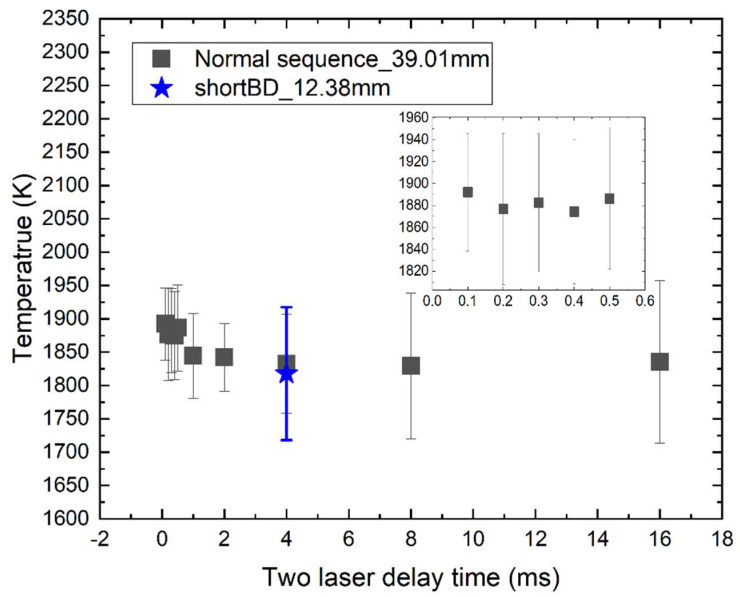
Temperature measured by dual-laser-induced breakdown thermometry (DLIBT) at the hot flue gas with a temperature of around 1750 K as a function of two-laser delay time. Black square: the far-away laser is firstly generated (i.e., Normal sequence) with a distance between breakdown spots of 39.01 mm; the blue star: the distance between breakdown spots is 12.38 mm. The inset shows the zoom-in data between 0 and 0.6 ms.

To evaluate the influence of the delay time between laser pulses on the accuracy of the thermometry, the delay time was varied from 100 µs to 16 ms. Figure 5 shows the measured gas temperature as a function of delay time. When the delay time is too short, the derived local gas temperature can be overestimated. For example, when the delay time varied from 100 µs to 0.4 ms, the derived temperature is around 30 K to 60 K larger than that of longer delay time. This temperature overestimation can be attributed to two reasons. First, with a short delay time, when the acoustic wave from Spot 2 travels to the microphone, it may pass through the heated region produced by the nearby laser-induced breakdown (Spot 1), and thereby the arrival time is decreased and eventually results in the higher temperature. Second, the acoustic wave signals from two breakdown spots can be superimposed and interfered, to make the time t_2_ in Figure 2 hardly distinguished. Therefore, the two-laser delay time should be larger than 0.5 ms to ensure the accuracy of this technique. It means that the DLIBT system can realize a temporal resolution of 0.5 ms. It should be noted that when the two-laser time delay is large (e.g., 16 ms), the error bar increases, possibly due to the flame unsteadiness and temperature fluctuation. 

The distance between the two breakdown spots is important since it concerns the spatial resolution. In the experiment, two distances, i.e., 12.38 mm and 39.01 mm, have been chosen and tested. As the outlet size of the burner is 60 mm×100 mm and the region with uniform temperature and species composition is around 40 mm in the central region, 39 mm is nearly the maximum value to obtain accurate temperature. The short distance of 12.38 mm is limited by the setup as the two focusing lenses are put close to focus the two laser beams in parallel. A closer distance between breakdown spots needs changing the optical paths. For comparison, Figure 5 shows a derived temperature with a distance of 12.38 mm at the delay time of 4 ms. It indicates that the longer distance between two laser breakdown spots (i.e., 39.01 mm) gives a smaller uncertainty than that of shorter distance (i.e., 12.38 mm), and the derived temperatures at two distances are almost the same under the same flame condition, which are 1833 K and 1818 K, respectively. The short distance of 12 mm means a Δt of around 13 μs. The shorter distance can result in much larger uncertainties of Δt. Therefore, it is better to choose the long distance between two breakdown spots (39.01 mm) to reduce the uncertainty in the current setup. However, the long-distance means a low spatial resolution. High spatial resolution is always needed when the temperature distribution is not perfectly uniform. The current system can give a spatial resolution at a level of around 10 mm.

**Figure 6:**
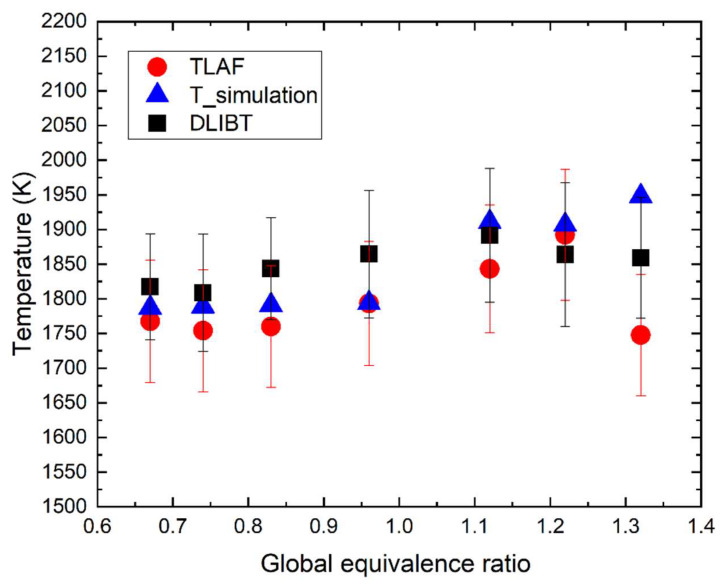
Temperature derived by DLIBT, TLAF, and CHEMKIN simulation versus the global equivalence ratio with the hot fuel gas temperature around 1800 K.

The hot flue gas with varying gas composition and temperature was employed to verify the DLIBT. Figure 6 shows the temperature of the hot flue gas derived by DLIBT, TLAF, and CHEMKIN simulation, respectively. The hot flue gas with a similar temperature of 1800 K was provided by flames with varying global equivalence ratios. To calculate the time of flight between two laser-induced breakdown spots, CHEMKIN simulations corresponding to the experimental conditions in Table 1 were performed to estimate the datasets of local species compositions, etc. At a given equivalence ratio of 0.67, 0.74, 0.83, 0.96, 1.12, 1.22, and 1.32, using the derived sound speed, the gas temperature can be derived based on Equation (4) in DLIBT. The averaged values are determined to be 1837.5 K, 1828.7 K, 1863.9 K, 1884.8 K, 1907.6 K, 1883.9 K, and 1879.4 K. The corresponding results by TLAF are 1770 K, 1750 K, 1760 K, 1790 K, 1840 K, 1890 K, and 1750 K. The adiabatic flame temperatures from CHEMKIN simulation are 1787 K, 1789 K, 1791 K, 1794 K, 1911 K, 1907 K, and 1948 K, respectively. In the oxidizing environments, the DLIBT gives a slightly higher value than that of simulation for 15–50 K. In the reducing environments, the temperature derived by DLIBT is slightly lower than the adiabatic flame temperature for 5–60 K. The results from DLBT and TLAF have a good agreement with large overlaps within their standard deviations. 

**Figure 7:**
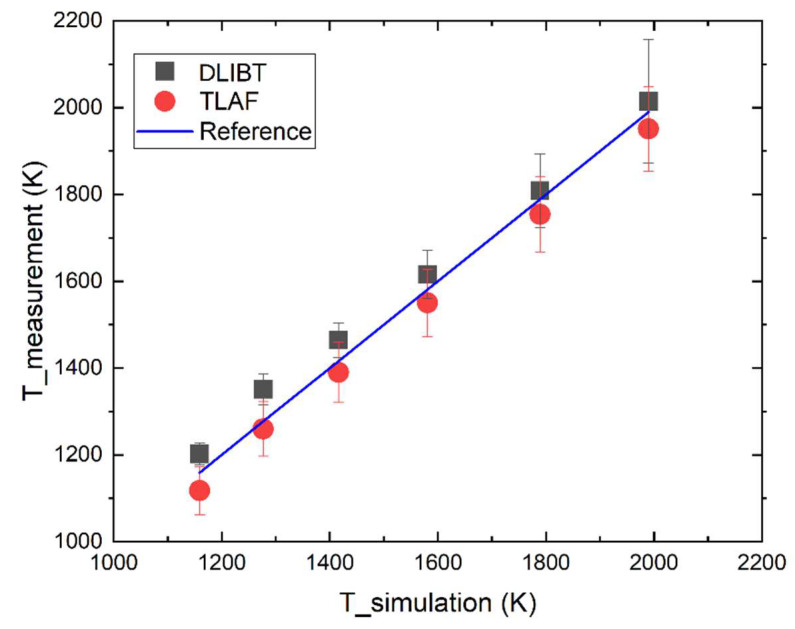
Temperature derived by DLIBT and TLAF versus the simulation temperature under different hot flue gas temperatures. The simulation temperature by CHEMKIN is set as the reference.

Figure 7 shows the temperatures of the hot flue gases with different temperatures, measured through DLIBT and TLAF versus the simulation temperature from CHEMKIN. Figure 7 shows excellent agreement between the temperatures measured by DLIBT and TLAF. A slightly systematic deviation can be observed, in which the temperatures obtained through DLIBT is about 40–50 K higher than the simulation temperature and 80–90 K higher than that through TLAF. 

The thermometric technique of TLAF was estimated to have an accuracy of 2.7% for averaged measurements and a precision of 1% in the experiment under given flame conditions. The results by TLAF are in good agreement with CARS measurements [9,17]. The derived temperature from DLIBT is precise and accurate, which is verified by the TLAF thermometry. The mean values agree well, and the standard deviation is almost the same for both methods. The uncertainty of the DLIBT is dominated by the uncertainties of the measurement of the distance between two breakdown spots and the determination of species compositions.

The proposed DLIBT increases the complexity of the system by using two individual lasers but it truly increases the spatial and temporal resolutions, compared to Lee’s method [14]. More importantly, the temperature derived by this technique can be up to 2200 K and it is not sensitive to the acoustic signal intensity and the laser pulse energy even when the pulse energy is larger than the breakdown threshold. Furthermore, the fluctuation of laser pulse energy and the time jittering influences the temperature measurement marginally and thus the cheap pulse laser can be used. Meanwhile, in the experiment, the laser pulse energy is around 100 mJ. On the other hand, this technique also has limitations. As it uses the acoustic emissions from the laser-induced breakdown, this technique is not fully non-intrusive. The breakdown and acoustic propagation can be impacted by particles or liquid droplets in the flow and thereby this technique may not be well suitable in aerosol environments. The detected acoustic signal can also be disturbed by the local noise. So, this technique should be optimized in a noisy environment. The spatial and temporal resolutions are still low compared to other 2D and single-shot technique. For the cases with fast temporal variations or steep spatial gradient, it can only derive an average temperature.

## 5. Conclusions

In this paper, DLIBT, a spatiotemporal resolution improved laser-induced breakdown thermometry via local sound speed measurement is proposed. Hot flue gases provided by a multi-jet burner with a wide range of temperatures and gas compositions were introduced to verify this technique. It is found that the measured temperature by DLIBT is well consistent with that calibrated by TLAF. Furthermore, by using the new measurement procedure with two individual lasers, high temporal and spatial resolutions, which are about 0.5 ms and 10 mm, respectively, have been achieved. As this method is relatively simple and robust, it is suitable to measure the temperature of hot flue gases or hot non-reacting gas in a nearly non-intrusive way.

## Figures and Tables

**Table 1:** Summary of the flame conditions adopted in this experiment, where the temperature measurement was performed at 5 mm above the burner outlet using two-line atomic fluorescence (TLAF).

Flame Case	Gas Flow Rate (sl/min)					Global Equivalence Ratio, Φ	Gas Product Temperature (K)
	Jet-Flow		Co-Flow				
	CH_4_	Air	O_2_	N_2_	Air		
**T1O2**	2.95	19.20	2.09	6.84	7.09	0.78	1950
**T2O2**	2.66	17.34	1.89	10.83	7.74	0.74	1750
**T2O3**	2.66	17.34	1.89	14.21	4.38	0.83	1760
**T2O4**	2.66	17.34	1.89	18.60	0.00	0.96	1790
**T2O5**	3.04	17.11	1.86	13.95	0.00	1.12	1840
**T2O6**	3.14	15.53	1.91	12.09	0.00	1.22	1890
**T2O7**	3.23	14.16	1.93	9.30	0.00	1.32	1750
**T3O2**	2.47	12.23	2.58	18.97	8.90	0.70	1550
**T4O2**	2.28	11.89	2.26	22.69	9.83	0.67	1390
**T5O2**	2.09	10.90	2.07	26.50	10.66	0.63	1260
**T6O2**	1.71	8.91	1.69	26.92	10.25	0.60	1120
